# Supplementary material for: Multi‐dataset identification of innovative feature genes and molecular mechanisms in keratoconus
Source: J Cell Mol Med. 2024 Sep 19;28(18):e70079. doi: 10.1111/jcmm.70079 (PMC11412914; doi:10.1111/jcmm.70079)
Supplement: Supplementary file 2 — Material S2. [file JCMM-28-e70079-s002.zip › 7.GSEA analysis/3.DBNDD1/KEGG_TYROSINE_METABOLISM.html]

Details for gene set KEGG\_TYROSINE\_METABOLISM[GSEA]

|  || Dataset | DBNDD1.group.cls#h\_versus\_l |
| Phenotype | group.cls#h\_versus\_l |
| Upregulated in class | l |
| GeneSet | KEGG\_TYROSINE\_METABOLISM |
| Enrichment Score (ES) | -0.38594878 |
| Normalized Enrichment Score (NES) | -1.2695879 |
| Nominal p-value | 0.12941177 |
| FDR q-value | 0.69109714 |
| FWER p-Value | 0.974 |
Table: GSEA Results Summary

  

Fig 1: Enrichment plot: KEGG\_TYROSINE\_METABOLISM      
 Profile of the Running ES Score & Positions of GeneSet Members on the Rank Ordered List

  

| SYMBOL | TITLE | RANK IN GENE LIST | RANK METRIC SCORE | RUNNING ES | CORE ENRICHMENT || 1 | MAOB | na | 696 | 0.415 | 0.0498 | No |
| 2 | TAT | na | 2033 | 0.298 | 0.0615 | No |
| 3 | IL4I1 | na | 3376 | 0.237 | 0.0627 | No |
| 4 | ADH6 | na | 3734 | 0.222 | 0.0899 | No |
| 5 | AOC2 | na | 4742 | 0.188 | 0.0926 | No |
| 6 | PNMT | na | 5290 | 0.171 | 0.1056 | No |
| 7 | ADH1B | na | 5723 | 0.158 | 0.1198 | No |
| 8 | TPO | na | 7176 | 0.120 | 0.0982 | No |
| 9 | ALDH3B1 | na | 8402 | 0.096 | 0.0791 | No |
| 10 | GSTZ1 | na | 9544 | 0.078 | 0.0593 | No |
| 11 | FAH | na | 9982 | 0.072 | 0.0588 | No |
| 12 | TH | na | 11060 | 0.057 | 0.0374 | No |
| 13 | METTL2B | na | 11655 | 0.050 | 0.0288 | No |
| 14 | LCMT2 | na | 11949 | 0.048 | 0.0283 | No |
| 15 | GOT2 | na | 13748 | 0.032 | -0.0181 | No |
| 16 | LCMT1 | na | 14355 | 0.027 | -0.0309 | No |
| 17 | COMT | na | 15365 | 0.020 | -0.0566 | No |
| 18 | AOX1 | na | 15596 | 0.018 | -0.0601 | No |
| 19 | TRMT11 | na | 16740 | 0.011 | -0.0912 | No |
| 20 | DDC | na | 16986 | 0.009 | -0.0967 | No |
| 21 | ADH7 | na | 17985 | 0.003 | -0.1250 | No |
| 22 | MAOA | na | 19119 | -0.005 | -0.1567 | No |
| 23 | MIF | na | 19184 | -0.006 | -0.1576 | No |
| 24 | GOT1 | na | 19996 | -0.013 | -0.1788 | No |
| 25 | ADH5 | na | 20394 | -0.016 | -0.1874 | No |
| 26 | ALDH3A1 | na | 23496 | -0.049 | -0.2685 | No |
| 27 | METTL6 | na | 24249 | -0.060 | -0.2801 | No |
| 28 | HEMK1 | na | 26003 | -0.087 | -0.3159 | No |
| 29 | DCT | na | 26024 | -0.088 | -0.3017 | No |
| 30 | ALDH3B2 | na | 28095 | -0.133 | -0.3389 | No |
| 31 | HPD | na | 29676 | -0.178 | -0.3544 | Yes |
| 32 | ADH1A | na | 29956 | -0.187 | -0.3309 | Yes |
| 33 | ALDH1A3 | na | 31867 | -0.257 | -0.3426 | Yes |
| 34 | HGD | na | 31891 | -0.258 | -0.2998 | Yes |
| 35 | ADH4 | na | 32701 | -0.306 | -0.2714 | Yes |
| 36 | TYRP1 | na | 32829 | -0.316 | -0.2218 | Yes |
| 37 | ADH1C | na | 33609 | -0.380 | -0.1802 | Yes |
| 38 | TYR | na | 33779 | -0.401 | -0.1175 | Yes |
| 39 | AOC3 | na | 33896 | -0.416 | -0.0507 | Yes |
| 40 | DBH | na | 34100 | -0.443 | 0.0181 | Yes |
Table: GSEA details [plain text format]

  

Fig 2: KEGG\_TYROSINE\_METABOLISM      
 Blue-Pink O' Gram in the Space of the Analyzed GeneSet

  

Fig 3: KEGG\_TYROSINE\_METABOLISM: Random ES distribution      
 Gene set null distribution of ES for **KEGG\_TYROSINE\_METABOLISM**
